# Protocol for quality control screening of brain organoid morphology

**DOI:** 10.1016/j.xpro.2026.104423

**Published:** 2026-03-13

**Authors:** Ilaria Chiaradia, Jerome Boulanger, Sofie Blomberg Elmkvist, Martin Røssel Larsen, Madeline A. Lancaster

**Affiliations:** 1 MRC LMB, CB2 0QH Cambridge, UK; 2 Department of Biochemistry and Molecular Biology, University of Southern Denmark, 5230 Odense, Denmark

**Keywords:** Developmental biology, Stem Cells, Organoids

## Abstract

Neural organoids can exhibit variability in both tissue shape and tissue identity. Here, we present a pipeline for rapid, protocol-agnostic quality control screening of brain organoids based on their overall gross morphology. We describe a semi-automated image analysis of organoid size, shape, and texture from 2D bright-field imaging. We provide a reference dataset of brain organoids with complex morphology. We show how to integrate input and reference organoids and perform the unbiased sample selection by *k*-means clustering.

For complete details on the use and execution of this protocol, please refer to Chiaradia et al.^1^

## Highlights

• Instructions for bright-field imaging of neural organoids for morphological analysis
• Steps for semi-automated image analysis to select organoids based on morphology
• Training data of organoids with good morphology from different protocols
• Code for morphospace generation and supervised clustering analysis

## Abstract (editor highlights)

Publisher’s note: Undertaking any experimental protocol requires adherence to local institutional guidelines for laboratory safety and ethics.

## Graphical abstract

**Figure:**
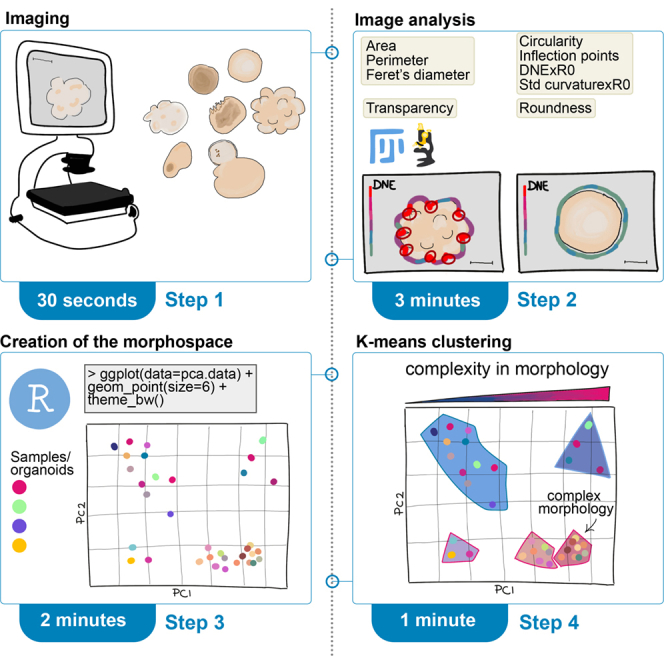

## Before you begin

Organoids vary in form and fate, and there is no perfect protocol for every scientific question. Neural organoids can refer to guided (or patterned) and unguided (or unpatterned) protocols.^2^ Guided organoids display a more consistent shape and forebrain identity, whereas unguided organoids exhibit greater batch-to-batch and intra-batch variability but display the coexistence of different brain regional identities and increased tissue structure complexity.^1^^,^^3^ Unguided organoids include cerebral organoids or brain organoids,^4^^,^^5^ where neural differentiation from the pluripotent embryoid body (EB) is obtained by default and the presence of intrinsic brain signalling centres – cortical hem, pallium-subpallium boundary – confers self-patterning features.^6^ Conversly, in guided, patterned, or regionalised neural organoids the intrinsic signalling is overruled by exogenous patterning with added growth factor inhibitors, to obtain more homogenous tissue identity. Forebrain identity is induced through the combined inhibition of both TFGß and BMP pathways in cortical spheroids (CSs)^7^ and forebrain organoids.^8^ Cortical organoids^9^ are obtained through TFGß, BMP, and WNT inhibition. Serum-free floating culture of embryoid body-like aggregates with quick reaggregation (SFEBq) are instead obtained with concomitant TFGß and WNT inhibition.^10^ Ventral forebrain organoids,^11^ region-specific choroid plexus organoids,^12^^,^^13^ hippocampal organoids,^12^ cerebellar organoids,^14^^,^^15^^,^^16^ and retinal organoids^17^^,^^18^ are other examples of central nervous system (CNS) guided organoids obtained by similarly inhibiting or activating relevant patterning pathways, and displaying varying morphologies. Previous works have focused on achieving reproducibility in size of organoids,^3^^,^^8^ but morphology has remained largely overlooked and no guidelines have been drawn so far for the morphological parameters to be expected in neural organoids. CSs are spherical small-sized organoids with dorsal forebrain mature features.^7^^,^^19^ Cerebral or brain organoids are heterogenous in shape, with occasional cystic tissue, and overall larger ventricles^1^^,^^4^ extending beyond the spherical perimeter of the organoid. Every ventricle, or neural bud, represents the working unit of the organoid as each one individually mimics the developing neural tube in cell type composition and tissue architecture.^1^^,^^4^

Morphology of neural organoids was shown to correlate with organoid macroarchitecture and transcriptional similarity to *in vivo* developing brain. This link was investigated on mature neural organoids at day 55.^1^ The description of the prediction of the transcriptional fidelity of different morphology organoids goes beyond the scope of this protocol and we redirect the reader to the previously published manuscript.^1^ Even an early organoid morphology screening (∼day 18) may predict tissue identity and transcriptional proximity to *in vivo,* thus informing on sample selection prior to downstream analysis on the mature organoid.

Here we describe the unbiased semi-automated quality control screening of neural organoids – both guided and unguided – based on their overall gross morphology. Gross morphology results from the combination of organoid size, shape, surface complexity, and texture. Our semi-automated tool complements existing image analysis software such as OrganoID and CellProfiler by focusing specifically on early prediction of organoid quality from 2D brightfield images based on size, shape, surface complexity, and texture. Unlike these general-purpose platforms, which compute broad morphological descriptors (e.g., solidity, eccentricity, circularity), our approach is calibrated on a curated reference dataset of brain organoids with complex morphology and emphasizes surface complexity metrics—such as curvature, Dirichlet Normal Energy (DNE), and inflection point density—that are not directly captured by CellProfiler or OrganoID. By quantifying these geometric parameters, our tool provides a complementary, organoid-specific framework for assessing morphological maturity and quality.

The protocol described here uses neural organoids at day 18. By this stage, neural organoids acquired stable and mature morphological features. Nevertheless, the protocol can be applied to organoids of different stages with due modifications. Previous work has shown that for < day 18 organoids, relevant morphological features are organoid size, roundness, and texture.^1^ Size is described by area, perimeter, and Feret’s diameter - the longest distance between any two points along the selection boundary. Roundness is the ratio of area on the squared major axis of the organoid. Texture is a readout of the transparency of the organoid measured as the mean response of the Laplacian of Gaussian (LoG) filter. By day 18, size and surface complexity of the organoid account for most of the variability in morphology.^1^ Surface complexity - measured as circularity, inflection points, Dirichlet Normal energy (DNE) x average radius (R0), and standard deviation of curvature (StdvCurvature) x R0 - describes the behaviour of the outer contour of the organoid and is a proxy for the number and size of ventricles bulging out of the perimeter of the organoid.^1^ Circularity is the ratio of area over squared perimeter. Inflection points describe the junction points between one neural bud and the next. Neural organoids with higher surface complexity were shown to have more ventricles of larger size and thicker neural progenitor layers.^1^ This advocates for a relevant role of surface complexity in describing organoid quality.

The pipeline described here enables the distinction of organoids with complex morphology from organoids with simpler morphology in a protocol-agnostic way. Both guided (SMAD+WNT inhibition) and unguided protocols were tested.^1^ The scope of this STAR Protocol goes beyond describing the specific protocols used to generate organoids; instead it focuses on how to quality-check samples according to their morphology. The applicability broadens to organoids other than neural, when the reference dataset is adjusted accordingly. We envision the pipeline can be applied to intestinal organoids^20^^,^^21^ where cysts and epithelial buds make the organoid suitable for surface analysis. Screening according to surface complexity might fail for organoids intrinsically round and smooth as it is the case for midbrain organoids.^22^ However, some degree of symmetry breaking can be observed during midbrain organoid maturation. A screening for surface complexity across time points could result in significant changes. For organoids with inherently smooth morphologies, a focus on size, geometry, and texture descriptors is advisable.

### Innovation

Until recently there have been no existing protocols for an unbiased and universal selection of neural organoids within quality standards, and consensus on quality criteria has been lacking. Samples are often hand-picked based on the experimenter experience. This contributes to inter-sample variability observed within batches and across batches in downstream analysis. Based on Chiaradia, Imaz-Rosshandler et al., 2023,^1^ we developed an image analysis pipeline for 2D brightfield images that focuses on organoid size, shape, surface complexity, and texture. Unlike conventional image analysis platforms that rely on manual selection of morphological descriptors, our approach is calibrated on a curated reference dataset of brain organoids with complex morphology and focuses on metrics of surface complexity that predict the number of neural ventricles. Chiaradia, Imaz-Rosshandler et al., 2023^1^ highlighted the link between organoid gross morphology and organoid quality, as organoids with complex morphology more closely mimic the transcriptional profile of the *in vivo* developing brain. Here we expand the description of the morphometric analysis, include a reference dataset, and provide raw images and codes in a clear and accessible way.

### Institutional permissions

Human ESCs (H9, female), chimpanzee male iPSC lines generated from Gallego Romero et al., 2015^23^ (Coriell ID #: S003649, S008861) were used in this study. H9 (WA09, WiCell) was purchased from WiCell. Human ESCs used in this project were approved for use in this project by the UK Stem Cell Bank Steering Committee and approved by an ERC ethics committee and are registered on the Human Pluripotent Stem Cell Registry (hpscreg.eu). Permission to use ESCs needs to be acquired from the relevant local and national institutions.

## Key resources table

**Table:** 

REAGENT or RESOURCE	SOURCE	IDENTIFIER
**Chemicals, peptides, and recombinant proteins**		

Matrigel	Corning	Cat#356234
FGF2 (FGF)	Peprotech	Cat#100-18B
SB 431542 (TGFβ inhibitor)	Sigma-Aldrich	Cat#S4317
Dorsomorphin (BMP inhibitor)	Sigma-Aldrich	Cat#P5499
IWR1endo (WNT inhibitor)	Stratech	Cat#S7086-SEL
CHIR 99021	Tocris	Cat#4423
EGF	R&D systems	Cat#236-EG

**Critical commercial assays**		

STEMdiff™ Cerebral Organoid Kit	StemCellTechnologies	Cat#08570

**Deposited data**		

Raw images of day 18 organoids of reference dataset	This paper	Zenodo Data: https://zenodo.org/records/16311731?preview=1&token=eyJhbGciOiJIUzUxMiJ9.eyJpZCI6IjBmNDU1YjY0LTAwNzUtNDY0Ny04MjdjLTMwN2UzMjFkZjIxOCIsImRhdGEiOnt9LCJyYW5kb20iOiI3MTQ4MzFkNWRiOGM2ZWU0ZDA4MmVmODc5MTM4ZWNmOCJ9.oRuLeB9xVoDqS6-3sYq30VMc0oGUrZ_XJSWhZ-acRcuZ8L1CEejmWdxBo8PSWwFV9IV2_N-hOszV_Fstg5iMBA
Raw images of day 18 input organoids	This paper	Zenodo Data: https://zenodo.org/records/16312269?preview=1&token=eyJhbGciOiJIUzUxMiJ9.eyJpZCI6IjEzY2IwMjI1LWI3NjEtNGFmMi1hNzY5LTM5NGI4OWUzYmQ3ZiIsImRhdGEiOnt9LCJyYW5kb20iOiI1M2ZkZDlmNjEyZjlkMjNmZDdmNjhlYWNmYmFlY2YzNSJ9.AzeNBWO2UlekRC_dXMBbKyBULPkFeVWIu8ZL3QwEwHfcW1y9KE02sgpCc2EDV52kJzMaeTiQVX05OcBcE43yNA
Measurement of morphological parameters of organoids in the reference dataset	This paper	Zenodo Data: https://zenodo.org/records/16311731?preview=1&token=eyJhbGciOiJIUzUxMiJ9.eyJpZCI6IjBmNDU1YjY0LTAwNzUtNDY0Ny04MjdjLTMwN2UzMjFkZjIxOCIsImRhdGEiOnt9LCJyYW5kb20iOiI3MTQ4MzFkNWRiOGM2ZWU0ZDA4MmVmODc5MTM4ZWNmOCJ9.oRuLeB9xVoDqS6-3sYq30VMc0oGUrZ_XJSWhZ-acRcuZ8L1CEejmWdxBo8PSWwFV9IV2_N-hOszV_Fstg5iMBA
Measurement of morphological parameters of input organoids + pcadata	This paper	Zenodo Data: https://zenodo.org/records/16312269?preview=1&token=eyJhbGciOiJIUzUxMiJ9.eyJpZCI6IjEzY2IwMjI1LWI3NjEtNGFmMi1hNzY5LTM5NGI4OWUzYmQ3ZiIsImRhdGEiOnt9LCJyYW5kb20iOiI1M2ZkZDlmNjEyZjlkMjNmZDdmNjhlYWNmYmFlY2YzNSJ9.AzeNBWO2UlekRC_dXMBbKyBULPkFeVWIu8ZL3QwEwHfcW1y9KE02sgpCc2EDV52kJzMaeTiQVX05OcBcE43yNA
Fiji/ImageJ code for morphological screening	This paper	Github Data: https://github.com/jboulanger/Organoid_morphology

**Experimental models: Cell lines**		

Human ESCs H9	WiCell	WA09
Chimpanzee male iPSCs	Coriell	S003649, S008861

**Software and algorithms**		

Morphological analysis of organoids from brightfield images	This paper	Github Data: https://github.com/jboulanger/Organoid_morphology
Fiji/ImageJ	Schindelin et al., 2012^24^	https://imagej.net/Fiji
R Studio v2023.06.0+421	R Core Team, 2018	http://www.R-project.org/

## Materials and equipment

EVOS XL Core Imaging System was used to image organoids in brightfield mode. Image analysis was performed on a MacBook Pro with 128 GB RAM and 1 TB of storage.

## Step-by-step method details

### Imaging

**Timing: 30 s**

We describe how to perform brightfield imaging of neural organoids with microscope settings ideal for downstream image analysis. We provide a code for batch calibration of organoid raw images to black and white 8-bit TIFF images with the same scale bar.

1. Image day 18 organoids in brightfield mode using an Evos XL Core (Thermo Fisher Scientific).
  ***Note:*** Maintain organoids in ultra-low attachment 6 well plates (Corning, 3471) in static culture at this early stage. According to the protocol used, organoids may or may not be embedded in Matrigel (Corning, 356234). Imaging can be done from 96 well plate for early time point organoids (COs < day 10) or compatibly with the size of the organoid.
  **CRITICAL:** Imaging setting must be kept consistent throughout the experiments. Refrain from imaging organoids at the edge of the well as the luminosity may differ. Avoid having more than one organoid on the field of view (Figure 1A). Choose the lightning mode that saturates upon dim lighting. Discard saturated and out-of-focus images (Figure S1A). Discard wells with bubbles in the medium or eliminate bubbles before imaging.
  a. Take one image with scale bar for reference.
    i. Scale bar can be removed or retained from the rest of the data-pool of images. It will not affect the final readout.

**Figure 1:**
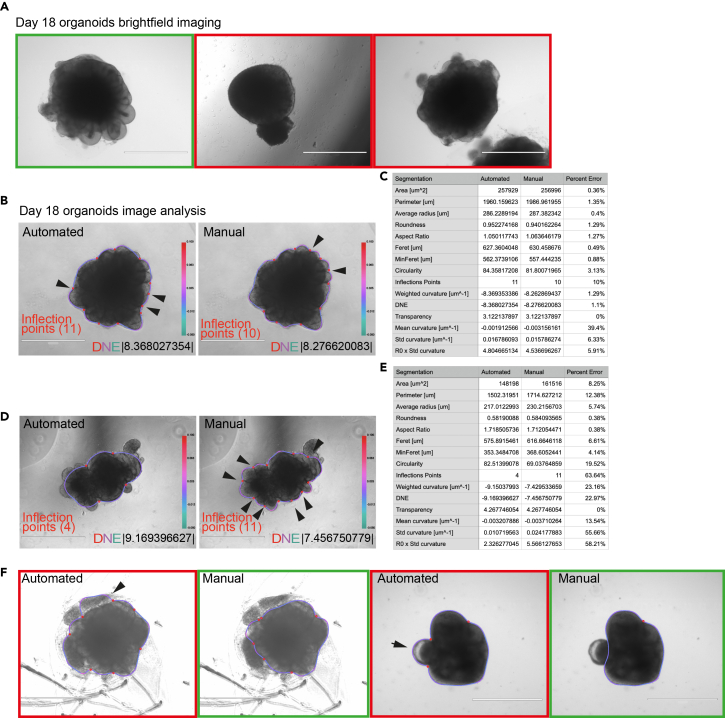
Organoid imaging and image tracing (A) Representative images of day 18 cerebral organoids (COs) imaged in brightfield mode using EVOS XL microscope. The image on the left (green frame) represents a good quality image selected for downstream image analysis. The remaining panels with red frames are to be excluded due to poor lightening or multiple organoids present in the same field of view. Scale bars: 1000 μm. (B–E) Image analysis of day 18 organoids ran through the morphometric analysis pipeline, choosing either the automated or the manual segmentation option. These options can vary in readout according to the sample analysed. Whereas in b the automated segmentation performs well, with a difference in the detected inflection points in automated versus manual of 1 and DNE differing by 0.09 and an overall low percent error (C), the organoid in d fails at the automated segmentation. The ROI traced automatically leaves out some ventricles (arrowheads), the inflection points are significantly less, the DNE differs of almost two points between manual and automated segmentation, and the percent error reaches peaks above 50% (E). Automated versus manual selection must be checked case by case. Scale bars: 1000 um. Scale bar indicates DNE (Dirichlet Normal Energy). (F) Representative images of automated segmentation of organoids. On the left, non-neural tissue (arrowhead) is included in the ROI during the automated segmentation. Similarly, cystic tissue is included in the ROI of the right panel organoid (arrow). Here manual segmentation is required for a supervised exclusion of these confounding tissues. Green frames are good segmentations; red frames indicate suboptimal segmentations. Scale bar: 1000 μm.
2. Calibrate the images using the following script on ImageJ/Fiji. Images have to be calibrated and in 8-bit TIFF format. Each TIFF image generated from brightfield imaging and converted to 8-bit weights 1.2MB.
  a. Download the latest version of ImageJ/Fiji from (https://fiji.sc, https://imagej.net/ij/download.html).
  b. Open Fiji and go to File->New->Script.
  c. Select the language from the header Language->IJ1Macro.
  d. Copy-paste the following script:

#@File(label="Input file") filename

#@Float(label="Pixel size", value = input) pixel_size

#@String(label="Unit", value="micron") unit

#@String(label="Pixel type", choices={"8-bit","16-bit","32-bit"}) pixel_type

/∗

∗ Set Pixel Size

∗

∗ Set the pixel size and type for the image and overwrite the image.

∗

∗ Warning this will update metadata.

∗

∗/

setBatchMode(true);

open(filename);

run("Set Scale...", "distance=1 known="+pixel_size+"

unit="+unit);

run(pixel_type);

saveAs("TIFF", filename);

print(filename);

close();

setBatchMode(false);

***Note:*** The script can be run in Batch mode, thus selecting multiple.tiff images at the same time for calibration. Images can be selected at the same time through ‘Add files’ command or the entire content of a folder can be selected through ‘Add folder content’ command.

**CRITICAL:** This will update the original image and the metadata. The original image will be replaced by the new 8-bit TIFF image in the same directory. In order to avoid overwriting the raw data, we recommend making a new folder with the relevant files for analysis.

### Image analysis

**Timing: 3 min**

We provide the ImageJ/FiJi code for the morphometric analysis of brightfield 2D neural organoid images capturing organoid size, shape, surface complexity, and texture. We describe how to perform batch organoid segmentation in manual/automated mode.

3. Analyse the calibrated images either on single or batch mode following the provided ImageJ/FiJi script. The analysis is a classical image analysis accessible to every user with the latest version of ImageJ/FiJi available open source. Open FiJi. In the toolbar select Plugins -> New -> Macro.
  a. Go to Github Data: https://github.com/jboulanger/Organoid_morphology Organoid_Morphology.ijm file, copy and paste the macro into the script editor and select language IJ1Macro.
  b. Press either Run or Batch for either one or multiple sample run respectively.
  c. Select output folder, namely where the outcome files will be saved.
    ***Note:*** The input file must be a TIFF file with a valid pixel calibration in microns.
  d. Select manual selection if a manual segmentation is preferred. Trace the contour using the polygon tool and close the selection.
    **CRITICAL:** We advise to run a manual segmentation for the beta testing of the pipeline onyour organoid type. We recommend checking the .jpg output files for accuracy of the traced ROI (Figures 1B and 1D).
    ***Note:*** The success of automated segmentation varies according to the complexity of the sample. Automated/manual segmentation error range varies from sample to sample and across the morphological parameters measured. The average percent of error between the automated and manual segmentation across all the parameters goes from 4.88% (Figure 1C) in a good automated segmentation to 19.64% in a poor automated segmentation (Figure 1E).
    **CRITICAL:** Manual segmentation might be required for organoids with non-neural tissue (e.g.: choroid plexus or fluid-filled cysts,^13^ neural crest or highly migratory neuron clumps) (Figures 1F and S1B).
  e. Choose what to display in the .jpg output image with the commands ‘overlay, display info, add colorbar, colorbar min, colorbar max, marker scale’.
    ***Note:*** Overlay: select the measure to overlay with the image (DNE-Dirichlet Normal Energy, Curvature).
    Display Info: select this to display additional information as an overlay on the image.
    Add colorbar: select this to display a color bar of the selected measure.
    Colorbar min: set the minimum value of the colorbar.
    Colorbar max: set the maximum value of the colorbar.
    Marker scale: define the scale of the marker for the inflection points.
  f. Save image as jpeg and close: select this to save the image as a .jpg file with the annotations (Curvature/DNE, additional info, colorbar).
    **CRITICAL:** ROI can be saved as filename.roi, amended, and reused. The image contour .roi file weights 2KB. Use Saved ROI: select this to use previously stored ROI (filename.zip -> filename.roi) in automated segmentation. For manual segmentation, previously saved ROI will be loaded automatically, the outline adjusted, and the roi manager updated.
4. For a sample pool of several images run the macro using the ‘Batch’ mode and save .jpg and .roi files. Each .jpg output file has a weight in the order of hundreds KB.
  a. Inspect .jpg output files to identify the poorly segmented samples (Figures 1B and 1D) and correct those with a manual segmentation step to update the .jpg and .roi files.
  b. Run again the macro on those files with the option ‘Use Saved ROI’ enabled.
  c. Save the .csv output table.

### Creation of the morphospace

**Timing: 2 min**

We describe the meaning of each parameter measured in the morphometric analysis. We provide instructions on how to organise the .csv file resulting from the image analysis for PCA analysis. We provide the R code for PCA analysis or morphospace of the samples.

5. Save the output of the image analysis results in a data sheet as a .csv file. The following information is displayed:

**Table:** 

File
Pixel size [μm]
Area [μm^2^]
Perimeter [μm]
Average radius [μm]
Roundness
Aspect Ratio
Feret [μm]
MinFeret [μm]
Circularity
Inflections Points
Weighted curvature [μm^−1^]
DNE
Transparency
Mean curvature [μm^−1^]
Std curvature [μm^−1^]
R0 x Std curvature

The meaning of each parameter is as follows: Pixel size: size of one pixel in μm; Area: area of selection in μm^2^; Perimeter: length of the outside boundary of the selection in μm; Average radius (R0): average radius throughout the ROI; Roundness: 4 x [Area/π (Major axis)2]; Aspect Ratio: ratio of width to height of the ROI; Feret’s diameter: the longest distance between any two points along the selection boundary; Min Feret’s diameter: the smallest distance between any two parallel lines tangent to the object’s boundary, measured at any angle; Circularity: 4π x (Area/Perimeter2); Inflection points: number of inflection points in the contour of the shape; Weighted curvature [μm^−^1]: sum of curvature values weighted by local segment lengths; Dirichlet Normal Energy (DNE): logarithm of the square of the variation of the normal n=(dy,-dx) of the contour projected on its tangent t=(dx,dy) where dx and dy are the first derivative in x and y^25^^,^^26^; Transparency: the mean response of the Laplacian of Gaussian (LoG) filter. The more the object is transparent the more textured it becomes and thus the higher the filter values; Mean curvature [μm^−^1]: average of the curvature values along the object’s contour; Standard Deviation of the Curvature (StdvCurvature): standard deviation of the logarithm of the sum of the square of the curvature of the contour. Where the curvature along the contour is the inverse of the radius of the tangent circle and is computed as [(dx ∗ dyy) – (dy ∗ dxx)]/[(dx∗dx) + (dy∗dy)] 3/2 where dx and dy are the first derivative in x and y and dxx and dyy are the second derivative of the contour; Standard Deviation of the Curvature (StdvCurvature) x R0: the standard deviation of the curvature is normalised by the average radius (R0).

6. Paste-transpose the data so as to have on the x axis the name of the samples and on the y axis the morphometric parameters. Save the file as.csv.
7. Select the parameters to include in the analysis.

***Note:*** Some parameters are redundant or relatively uninformative in terms of morphology and can be excluded, such as previously done in Chiaradia, Imaz-Rosshandler et al., 2023^1^ for pixel size, average radius, aspect ratio, min Feret’s diameter, weighted curvature, and mean curvature. Standard deviation of the curvature and DNE can be multiplied by the average radius (R0) to normalise on the organoid size.

8. Download R or R studio (https://posit.co/download/rstudio-desktop/).
9. Input in the editor the following code to perform a principal component analysis of the morphological parameters using the function prcomp:

library(ggplot2) # load or download ‘ggplot2’package.

library(writexl) # load or download ‘writexl’package.

library(corrplot) # load or download ‘corrplot’ package.

library(factoextra) # load or download ‘factoextra’ package.

example <- read.csv('/Users/add/Desktop/example.csv',

row.names=1) # opens the csv file in R.

data.matrix <- example # transforms the csv file in a matrix.

head(data.matrix) #to visualise the matrix.

rownames(data.matrix) <- paste(c("Area [umˆ2]", "Perimeter [um]", "Feret [um]", "Roundness", "Circularity", "Inflection Points", "DNExR0", "R0 x Std curvature", "Transparency"), sep="")

# specifies the heading of the matrix. Change it according to the parameters included in the .csv file.

head(data.matrix) # to visualize the data matrix.

pca <- prcomp(t(data.matrix), scale=TRUE) # prcomp function transforms the original data into a set of uncorrelated variables called principal components, ordered by the amount of variance they capture. scale=TRUE ensures that each feature is scaled to have unit variance before performing PCA.

plot(pca$x[,1], pca$x[,2]) # plots the first principal component (pca$x[,1] contains the first principal component scores) against the second principal component (pca$x[,2] contains the second principal component scores).

pca.var <- pca$sdevˆ2 # squaring the standard deviation gives the variance explained by each component (pca.var).

pca.var.per <- round(pca.var/sum(pca.var)∗100, 1) # computes the rounded percentage of the proportion of the total variance explained by each principal component.

barplot(pca.var.per, main="Scree Plot", xlab="Principal Component", ylab="Percent Variation") # creates a Scree Plot using a bar chart to visualize the percentage of variance explained by each principal component.

pca.data <- data.frame(Sample=rownames(pca$x),

X=pca$x[,1],

Y=pca$x[,2]) # creates a data frame where sample identifiers are included as the sample column, the scores for the first principal component (PC1) are stored in column X and the scores for the second principal component (PC2) are stored in a new column named Y.

pca.data # shows the pca data tab.

write_xlsx(pca.data, "/Users/…directory.xlsx") # generates a .xlsx file to be exported.

pca.data$name = rownames(pca.data) # extracts sample names from the pca data matrix.

ggplot(data=pca.data, aes(x=X, y=Y, color=pca.data$name)) +

geom_point(aes(color = pca.data$name), size = 6) +

xlab(paste("PC1 - ", pca.var.per[1], "%", sep="")) +

ylab(paste("PC2 - ", pca.var.per[2], "%", sep="")) +

theme_bw() # plots the data points in pca.data in the morphospace drawn through ggplot R package assigning a color to each point based on the values in the name column of pca.data and displaying PC1 on the x axis and PC2 on the y axis.

res.var <- get_pca_var(pca) # extracts the information on how variables contribute to the definition of principal components.

res.var$cos2 # the squared cosine values measure the quality of representation for each variable on the components.

corrplot(res.var$cos2, is.corr=FALSE, tl.col="black", tl.srt=45).

# to visualize the cos^2^ values of variables from the PCA analysis in a correlation plot using the ‘corrplot’ package. is.corr=FALSE is to specify that the input matrix is not a correlation matrix but rather a data matrix. Tl.col specifies the color of the labels, tl.srt the angle.

### *k*-means clustering

**Timing: 1 min**

We provide a reference dataset of complex morphology organoids to integrate with the user input sample data pool. We describe how to generate unbiased *k*-means clustering of reference and input samples. We describe how to interpret the position of samples in the morphospace and how to select complex morphology organoids from the input samples.

10. Add to the .csv file from Image Analysis of input organoids (bullet point 4) the reference dataset of complex morphology organoids extracted from Chiaradia, Imaz-Rosshandler et al., 2023^1^ and provided as ‘Measurement of morphological parameters of organoids in the reference dataset’ in the Key Resource Table-KRT.

**Figure 2:**
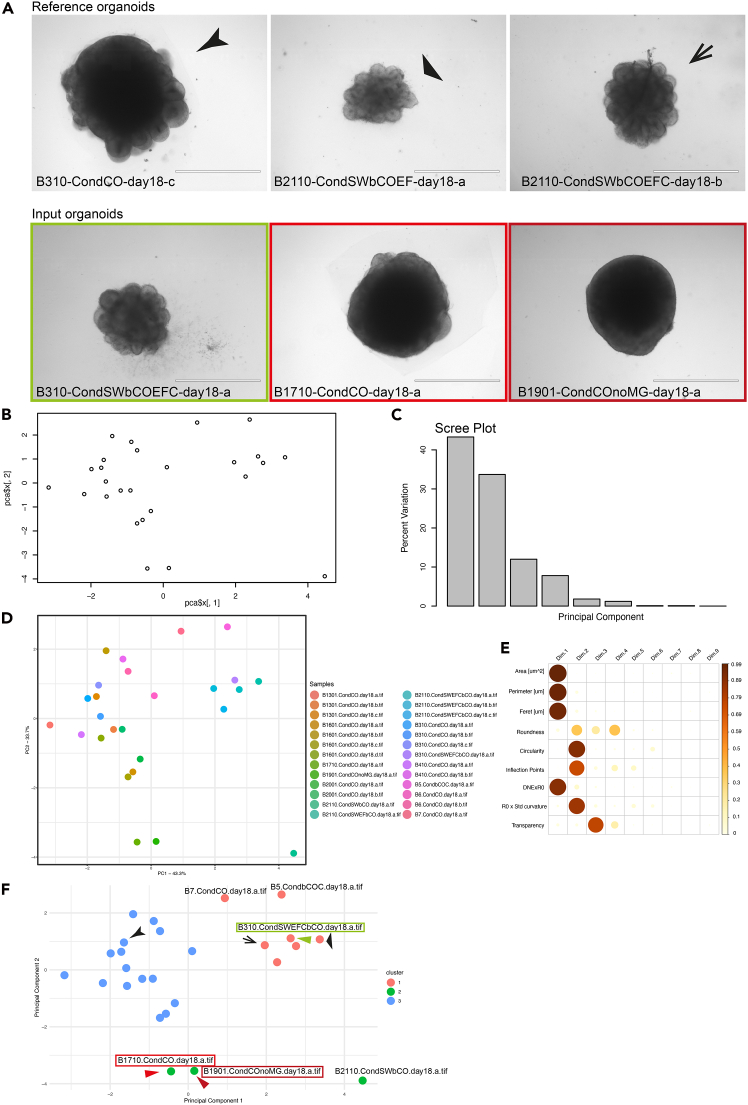
Organoid morphological analysis and *k*-means clustering (A) Upper panels – representative brightfield images of organoids included in the reference dataset (KRT). Organoids were generated with different protocols, as indicated in labels. Arrows and arrowheads refer to datapoints in Figure 2F. Organoids were imaged at day 18 in brightfield mode using an EVOS XL microscope. Scale bars: 1000 μm. Lower panels – representative brightfield images of input organoids tested for their morphological features against reference organoids. Organoids were generated with different protocols. Colored frames refer to datapoints in Figure 2F. Green frame refers to organoid that passed the morphology quality screening. Red frames are organoids that did not pass. Organoids were imaged at day 18 in brightfield mode using an EVOS XL microscope. Scale bars: 1000 μm. B=number of batch; CondCO – cerebral organoids made according to an optimized protocol with a proprietary media but lacking patterning molecules; CondSWbCO +EF – guided cerebral organoid treated with SMAD WNT (TGFß and WNT) inhibitors plus treatment with epidermal growth factor (EGF) and fibroblast growth factor (FGF) during neuroepithelial expansion^1^; SWbCO + EFC – guided cerebral organoid treated with SMAD WNT (TGFß and WNT) inhibitors plus treatment with epidermal growth factor (EGF), fibroblast growth factor (FGF), and GSK3 Inhibitor/WNT Activator (CHIR-99021) during neuroepithelial expansion; CondCO noMG (CO without the addition of Matrigel). (B) Raw PCA plot of reference + input organoids displaying PC1 percent variation on the x axis and PC2 percent variation on the y axis. (C) Screeplot of the percentage of variation in the morphospace explained by each principal component (vertical bars). (D) PCA plot of reference + input organoids color coded by sample displaying PC1 percent variation on the x axis and PC2 percent variation on the y axis. (E) Correlation plot of the PCA analysis shown in (D) showing the contribution of each morphometric parameter as the squared cosine across principal components. Color intensity and size of the circles are proportional to the contribution of each parameter to the principal components. (F) PCA plot with *k*-means cluster annotation where k=3. Arrowheads and colored frames refer to (A).

***Note:*** Day 18 reference organoids are selected based on heuristic morphological evaluation. The dataset includes samples from seven different batches and three different protocols (CO – cerebral organoids made according to an optimized protocol with a proprietary media but lacking patterning molecules^1^^,^^27^; SWbCO +EF – guided cerebral organoids treated with SMAD WNT (TGFß and WNT) inhibitors plus treatment with epidermal growth factor (EGF)/fibroblast growth factor (FGF) during neuroepithelium expansion^1^; SWbCO + EFC – guided cerebral organoids treated with SMAD WNT (TGFß and WNT) inhibitors plus treatment with epidermal growth factor (EGF)/fibroblast growth factor (FGF)/GSK3 Inhibitor/WNT Activator (CHIR-99021) during neuroepithelium expansion.^1^ Input organoids we test on the reference pool are generated according to the following protocols: CO, SWbCO + EFC, SWbCO, CO noMG (CO without the addition of Matrigel), bCO + C (unguided cerebral organoids in basalmedium^4^ plus GSK3 Inhibitor/WNT Activator (CHIR-99021) during neuroepithelium expansion^1^). For further details on the protocol steps refer to Chiaradia, Imaz-Rosshandler et al., 2023.^1^

**CRITICAL:** Original brightfield .tif images from each sample can be found in ‘Raw images of day 18 organoids of reference dataset’ and ‘Raw images of input organoids’ in the KRT. Representative images are shown in Figure 2A.

11. Perform the principal component analysis of the input samples plus the reference dataset as in step point 9 (Figure 2B).
12. Obtain the Scree Plot of the percentage of variance explained by each principal component (Figure 2C).
13. Plot the pca.data with ggplot function choosing the two most representative principal components (Figure 2D).

***Note:*** The distance of the input samples from the reference samples can be explained by looking at the corrplot showing the contribution of each variable for the definition of the principal components (Figure 2E). The variability across the morphospace may be explained by principal components other than PC1, PC2. Refer to troubleshooting Problem 4.

14. Perform *k*-means clustering with the following code:

**CRITICAL:** Ensure the data matrix in the .csv file has all variables scaled.

pca <- prcomp(t(data.matrix, scale. = TRUE)) # performs the pca analysis using the prcomp function.

summary(pca) # view PCA summary to understand variance explained.

explained_variance <- cumsum(pca$sdevˆ2 / sum(pca$sdevˆ2)) # computes the cumulative variance explained by each PC.

selected_components <- which(explained_variance > 0.9)[1] #retains enough PCs to explain ∼90% of the variance.

pca_data <- pca$x[, 1:selected_components] # selects topcomponents

set.seed(123) # by providing the same seed, the random number sequence generated is identical across multiple runs of the code.

k <- 3 # choose number of clusters (3).

kmeans_result <- kmeans(pca_data, centers = k, nstart= 25) # performs k-means analysis with k=3 and nstart to have different random centroids.

kmeans_result$cluster # shows the cluster attribution for each sample.

pca_df <- as.data.frame(pca_data).

pca_df$cluster <- as.factor(kmeans_result$cluster) # Creates a data frame with PCA data and cluster assignments.

ggplot(pca_df, aes(x = PC1, y = PC2, color = cluster)) + geom_point(size = 6) + xlab(paste("PC1 - ", pca.var.per[1], "%", sep="")) + ylab(paste("PC2 - ", pca.var.per[2], "%", sep="")) +

theme_bw() # Plots the first two PCs with cluster assignment.

***Note:*** The results show the position in the morphospace of the input samples (Figure 2F – labels) compared to the reference organoids. Random determination of k=number of clusters for *k*-means analysis might influence the selection of organoids to discard or retain. Refer to troubleshooting problem 5. Having set k=3 in *k*-means clustering, the result is a splitting of the reference dataset across clusters 1 and 3. Three input organoids cover entirely cluster 2 (B2110-CondSWbCO-day18-a, B1901-CondCOnoMG-day18-a, B1710-CondCO-day18-a). The remaining three input organoids (B7-CondCO-day18-a, B5-CondbCOC-day18-a, B310-CondSWEFCbCO-day18-a) fall in cluster 1 together with some reference organoids. Based on sample clustering, these last three samples pass the morphology-based quality check and can be retained for further downstream analysis. Although PC1 explains most of the variance observed across samples (Figure 2D), and B1901-CondCOnoMG-day18-a, B1710-CondCO-day18-a samples fall on the same side of the PC1 axis of the majority of the reference dataset (cluster 3), PC1 is mainly represented by organoid size descriptors (Figure 2E). Organoid size per se does not necessarily correlate with organoid quality and regional identity. Instead, surface-to-volume ratio can influence the representation of pure neural tissue.^28^ Suface-to-volume ratio in our morphological analysis is described by Circularity. Circularity emerges in PC2 (Dimension 2), alongside inflection points and Std curvature x R0. These last two factors describe organoid surface complexity, meant as the number and size of neural buds bulging out of the organoid perimeter. This is a good proxy for the amount of neural tissue in the organoid. The definition of complex morphology relies on surface complexity – high circularity, inflection points, Dirichlet Normal energy (DNE), and standard deviation of curvature. In the linked manuscript (Chiaradia I, Imaz-Rosshandler I, Nilges BS, Boulanger J, Pellegrini L, Das R, Kashikar ND, Lancaster MA. Tissue morphology influences the temporal program of human brain organoid development. Cell Stem Cell. 2023 Oct 5;30(10):1351-1367.e10. https://doi.org/10.1016/j.stem.2023.09.003.), we demonstrated that day 18 organoids with higher surface complexity displayed more and larger ventricles with increased proliferative layer thickness. These traits suggest a high organoid quality. We further showed that day 55 high score morphology organoids, resulted from supervised *k*-means clustering, performed better in matching the *in vivo* foetal brain transcriptome of three published datasets. In conclusion, separation along PC2 axis can predict organoid quality. This suggests to retain input organoids B7-CondCO-day18-a, B5-CondbCOC-day18-a, B310-CondSWEFCbCO-day18-a and to exclude input organoids of cluster 2 (Figure 2F).

***Note:*** Organoids from different protocols, both from the reference dataset and from input samples, fall in the same morphology cluster (black arrows Figures 2A and 2F). On the other hand, organoids from the same protocol (B310-CondCO-day18-c arrowhead left panel Figure 2A and in Figure 2F; B1710-CondCO-day18-a red frame middle panel Figure 2A and in Figure 2F) can display different morphologies.

## Expected outcomes

### Protocol influence on organoid morphology

**Figure 3:**
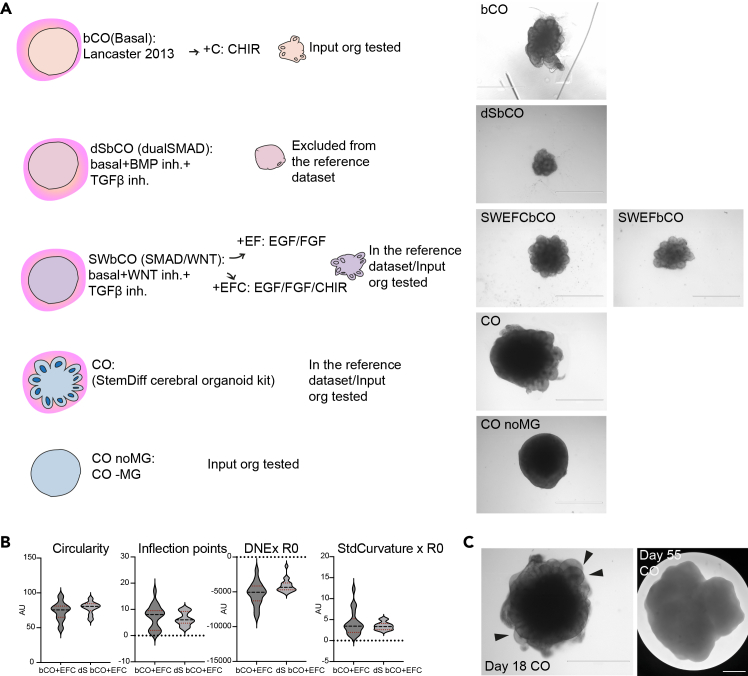
Organoid size and surface complexity across different protocols (A) Cartoon of the main subtypes of neural organoids developed according to unguided (bCO, CO) or guided (dS, SW) protocols. Specification on whether the organoid condition has been included in the reference dataset and/or used as a test input organoid. Right, representative brightfield images of day 18 organoids from the listed conditions. Organoids display differences in size and surface complexity. Scale bars: 1000 μm. (B) Distribution of circularity, inflection points, DNE x R0, and StdCurvature x R0 for day 18 bCOs and dSbCOs treated with EGF/FGF/CHIR. Median and quartiles are shown as dotted lines. Six batches per condition were analysed. Graphs from Figure S1 of Chiaradia, Imaz-Rosshandler et al., 2023. (C) Representative brightfield images of day 55 and day 18 COs showing different size and surface complexity. Ventricles, clearly visible at day 18 (arrowheads), disappear in the outer contour of day 55 organoid. Scale bars: 1000 μm. CO – cerebral organoids made according to an optimized protocol with a proprietary media but lacking patterning molecules; CO noMG – CO without the addition of Matrigel; SWbCO – guided cerebral organoid treated with SMAD WNT (TGFß and WNT) inhibitors; dSbCO – guided cerebral organoid treated with dual SMAD (dS) (TGFß and BMP) inhibitors; bCO – unguided cerebral organoids in basal medium; EFC – EGF, FGF, CHIR.

As observed in Chiaradia, Imaz-Rosshandler et al., 2023,^1^ protocol-to-protocol variations are expected to influence organoid morphology. Specifically, dualSMAD (dS) – TFGß+BMP inhibition produces smaller organoids with spherical shape and overall smoother surface^1^ (Figure 3A). SMAD+WNT inhibition gives smaller organoids compared to COs (cerebral organoids made according to StemDiff cerebral organoid kit StemCell Technologies Cat#08570), yet comparable in size to dS. However, SW (SMAD+WNT inhibition) organoids display an overall degree of surface complexity comparable to COs. Unguided cerebral organoids in basal medium^4^ (bCOs) display highly variable size and surface complexity. Guided dS organoids are more consistent in shape compared to unguided bCOs^1^ (Figure 3B). The observed difference in size across protocols occurs despite the same number of pluripotent stem cells to start with in the embryoid body (2000 cells). COs without Matrigel (noMG) are similar in size to COs but with smoother surface at day 18 (Figure 3A).

We demonstrate that our morphological screening works across a variety of methods to generate forebrain organoids and the reference dataset includes both guided and unguided samples (COs and SWbCOs). We excluded dSbCOs from the reference dataset as they display consistent low-grade morphology and surface complexity. We excluded CO noMG for the same reason. We suggest taking this aspect into account when applying this pipeline to dS samples. Guided dS and SW organoids might occupy a different K-means cluster than unguided COs due to their smaller size. We advise to control for this while running the analysis (Figure 2F) and to give priority to surface complexity parameters as the main rulers of organoid morphology (Troubleshooting Problem 3). We expect the pipeline to perform better on single-protocol organoids and on protocols represented in the reference dataset. We expect the screening to fail with intrinsically simpler shaped organoids, such as midbrain organoids^22^ or organoids with very different morphology, such as retinal organoids.^29^

### Young organoids are more suitable than mature organoids for morphological screening

Although the morphological screening was previously applied to both day 18 and day 55 organoids,^1^ in our experience early organoids are better subjects for this analysis. Indeed, while day 55 organoids appear overall rounder in shape and ventricles are not always visible from brightfield imaging, day 18 organoids display higher transparency and neural buds are not covered by migrating neurons (Figure 3C arrowheads). At day 18 the transparency of neuroepithelium coexists with the darker core of the organoid. The difference in transparency value loses significance in day 55 organoids, when the organoid is too thick for light to penetrate the tissue (Figure 3C). We therefore suggest omitting transparency in later stage organoids.

### Generalizability of the tool to different time points and cell lines

Organoid protocol might require adapting the timing of media transitions and image capturing according to the cell line and the species. We further tested our morphological analysis on COs (cerebral organoids made according to StemDiff cerebral organoid kit StemCell Technologies Cat#08570) from two chimpanzee male iPSC lines generated from Gallego Romero et al., 2015^23^ (Coriell ID #: S003649, S008861) using the protocol for human ESCs H9^1^ but with the transition to neural induction medium at day 3 instead of day 5 and Matrigel embedding at day 5 instead of day 7. For the description of the protocol, we refer the reader to Chiaradia, Imaz-Rosshandler et al., 2023^1^ COs organoids. Manual image analysis on six day 9 organoids from two batches from two chimpanzee cell lines and further PCA plotting generated a morphospace with sample distance largely explained by PC1 (Figure S2A). PC1 (Dim 1) is composed of organoid size descriptors and parameters of surface complexity (Inflection points, DNExR0) (Figure S2B). As organoids from both cell lines display high surface complexity with several neural buds (Figure S2C), *k*-means clustering generated two clusters split along PC1, thus based on surface-to-volume ratio, taking into account both size and surface descriptors. The two clusters correspond to the two different cell lines (Figure S2D). This analysis, although limited in sample number, demonstrates the generalizability of our morphometric pipeline to different cell lines, species, and time points.

The tool was further used by external users in the context of testing the effect of Matrigel exposure on organoid morphology. Cerebral organoids from ESCs H9 generated according to StemDiff cerebral organoid kit (StemCell Technologies Cat#08570) in three batches were treated with Matrigel at day 7 (+MG) or cultured without Matrigel (-MG) (Figure S2E). Samples were imaged at day 11 and further processed through the morphological screening pipeline described above. Matrigel treatment overwrote batch-to-batch differences as shown in the morphospace (Figure S2F). Unbiased *k*-means clustering with k=2 revealed a segregation of samples based on +MG/-MG treatment evident as early as 96 hours post treatment (Figure S2G). +MG/-MG samples splitted across PC1 which contained size and surface complexity descriptors (Figure S2H).

The experiments demonstrate that our morphological screening pipeline is broadly applicable across different biological contexts, from organoid batch-to-batch variation, cell-line differences, treatment conditions, and protocol modifications.

## Quantification and statistical analysis

### Number of batches/conditions of the reference dataset

The reference dataset contains organoids from seven different batches and three different conditions: guided SWEFbCO (one organoid), SWEFCbCOs (three organoids), unguided COs (16 organoids). The total number of organoids in the reference dataset is 20. A bigger number of samples in the reference dataset may help discriminate good from poor morphology in input organoids. In addition, a greater representation of different protocols/conditions in the reference dataset may contribute to a broader applicability of the screening pipeline to different organoid types.

### Morphological parameters

We excluded from the PCA analysis the following parameters as not meaningful for our analysis: average radius, aspect ratio, minimum Feret’s diameter, weighted curvature, DNE (Dirichlet Normal Energy), mean curvature, standard deviation of the curvature. We suggest a sample-tailored selection of the relevant parameters.

## Limitations

Guided dSbCOs may fail the pipeline as they display higher homogeneity in tissue shape compared to the unguided conditions as shown by the distribution of circularity, inflection points, DNExR0, and StdCurvaturexR0 for bCOs and dSbCOs treated with EGF/FGF/CHIR (Figure 3B). Additionally, dSbCOs have an overall round shape with fewer ventricles extruding from the organoid profile (Figure 3A). This invalidates surface complexity descriptors. Whereas the algorithm might perform suboptimally for dSbCOs + unguided COs mixed sample pool, it may spot intrinsic sample-to-sample differences across all dSbCOs organoids. In this case, we advise to change the reference dataset to dS organoids accordingly. Excess of Matrigel, outgrowing neural and non-neural tissue can hamper organoid segmentation. We advise to adopt manual segmentation for challenging samples. For such biological pitfalls and imaging artifacts we point the reader to troubleshooting problems 1 and 2.

## Troubleshooting

### Problem 1

When imaging in brightfield mode, changing the setting of the light source across the imaging session, using unclean lenses, or imaging the organoid at the edge of the well might result in dark images (related to Step 1 [Imaging]).

### Potential solutions

Image in brightfield black/white mode. Choose the lighting mode that saturates upon the dimmer lighting percentage. Maintain the same imaging settings throughout the imaging sessions. Avoid imaging organoids at the edge of the well. Overly saturated images, blurred or out-of-focus images are to be discarded (Figure S1A).

### Problem 2

ROI tracing and morphological assessment is challenging because of highly migratory tissue covering the outer surface (related to Step 2 [Image analysis]). This tissue is thought to be either non-neuroepithelial tissue (Figure S1B arrows) or neuronal processes from directed neural differentiation (Figure S1B arrowheads). The problem occurs mainly upon Matrigel embedding. Matrigel offers a protein-rich substrate for cell and axon outgrowth.

### Potential solutions

• Use manual segmentation option and supervise the ROI tracing to exclude tissue outgrowth. Discard images where the organoid perimeter is entirely covered by migrating tissue.
• Use the same Matrigel batch throughout the experiment to avoid batch-to-batch variation in composition.
• Promptly remove Matrigel upon tissue overgrowth.

### Problem 3

Sample distribution across the morphospace can appear sparse without apparent clustering, even for organoids from the same batch, condition, and timepoint (related to Step 3 [Creation of the morphospace]). This might derive from an intrinsic sample-to-sample difference that can be confirmed by looking at the brightfield images or from an intrinsic limitation of the PCA analysis in dealing with low n pool of samples (Figure S1C).

### Potential solutions

• We recommend dealing with larger datasets to maximise the reduction in complexity operated by the PCA analysis. Here we show same-batch day 18 COs when only three samples are analysed and same-batch day 18 COs with n=6 organoids (Figure S1C left and middle panels).
• Same-batch/condition/time-point dataset might highlight sample-to-sample differences. Multiple-batch dataset might highlight batch-to-batch variability (Figure S1C right panel). Nevertheless, we showed that morphological sample-to-sample proximity can overrule batch-to-batch differences and that organoids from different conditions can perform equally well in terms of morphology.
• Some of the morphological parameters may not be informative or worse, misleading, according to the sample type. In our case, we demonstrated that organoid size is not reflective of tissue composition and that organoids with similar surface complexity can differ in size, especially across different batches (B1601-CondCO-day18-b (°), B7-CondCO-day18-a (∗), Figure S3A PCA plot, S3B correlation plot, and S3C cluster analysis). In such cases, size descriptors can be omitted from the morphospace (Figure S3D PCA plot, S3E correlation plot, S3F cluster analysis, S3G representative images of poor morphology and borderline organoids as labelled in F) or individual parameters can be plotted independently (Figure S3H). The analysis changes from an unbiased morphological screening to a selective parameter choice.

### Problem 4

The variability across the morphospace may only be partially explained by PC1 and PC2 (related to Step 4 [*k*-means clustering]). PC3 contributes to a significant percentage of the variation observed across the dataset, being very close to PC2 in percent variation (Figure S4A PCA plot, S4B correlation plot, S4C scree plot).

### Potential solutions

• The dataset can be expanded including a larger n=samples.
• If the scree plot shows relevant morphological parameters largely underrepresented in PC1 and PC2 (Dim1, Dim2), but present in PC3 (Dim3), the user can decide to plot PC1 versus PC3 or PC2 versus PC3. The PCs chosen are entirely sample-dependent and can be interchanged (Figure S4D PCA plot).
• The PCA plot can be computed as a 3D plot that includes PC1, PC2, PC3 (Figure S4E) computing the following code:

library(plotly) # Loads the required extra library

pca <- prcomp(t(data.matrix), scale=TRUE) # Performs the PCA

pca.var <- pca$sdevˆ2

pca.var.per <- round(pca.var / sum(pca.var) ∗ 100, 1) # Extracts the variance percentages for each PC

pca.data <- data.frame(Sample = rownames(pca$x),

X=pca$x[,1],

Y=pca$x[,2],

Z=pca$x[,3]) # Creates a dataframe with PC1, PC2, and PC3

fig <- plot_ly(data = pca.data,

x = ∼X,

y = ∼Y,

z = ∼Z,

text = ∼Sample,

type = "scatter3d",

mode = "markers",

marker = list(size = 6, color = as.numeric(factor(pca.data$Sample)), colorscale = "Viridis" )

) %>%

layout(title = "3D PCA Plot",

scene = list(xaxis = list(title = paste("PC1 -", pca.var.per[1], "%")),

yaxis = list(title = paste("PC2 -", pca.var.per[2], "%")),

zaxis = list(title = paste("PC3 -", pca.var.per[3], "%")))) # Creates an interactive 3D scatter plot using plotly

fig # Displays the plot

### Problem 5

Random determination of k=number of clusters for *k*-means analysis might influence the selection of organoids to discard or retain (related to Step 4 [*k*-means clustering]). In Figure 2F, we chose to input k=3 instead of k=2 as with two clusters only, poor morphology organoids (Figure 2F cluster 2; Figure S5A dashed contour and respective brightfield images) would end up clustering with good morphology organoids (Figure S5B dashed arrowheads and respective brightfield images).

### Potential solutions

• Test multiple *k*-means analysis with increasing k.
• Supervise the analysis by pinning three good and three low morphology samples and verify their distribution in the morphospace and across the clusters.
• Verify the composition of the PCs that describe the distance in space between the clusters.

## Resource availability

### Lead contact

Further information and requests for resources and reagents should be directed to and will be fulfilled by the lead contact, Madeline A. Lancaster (madeline.lancaster@mrc-lmb.cam.ac.uk).

### Technical contact

Questions about the technical specifics of performing the protocol should be directed to and will be fulfilled by the technical contacts, Ilaria Chiaradia (ilariachiaradia.bio@gmail.com) and Jerome Boulanger (jeromeb@mrc-lmb.cam.ac.uk).

### Materials availability

This study did not generate new unique reagents.

### Data and code availability

• The reference dataset for Figures 2 and S3–S5 in the paper is available at Zenodo Data: https://zenodo.org/records/16311731?preview=1&token=eyJhbGciOiJIUzUxMiJ9.eyJpZCI6IjBmNDU1YjY0LTAwNzUtNDY0Ny04MjdjLTMwN2UzMjFkZjIxOCIsImRhdGEiOnt9LCJyYW5kb20iOiI3MTQ4MzFkNWRiOGM2ZWU0ZDA4MmVmODc5MTM4ZWNmOCJ9.oRuLeB9xVoDqS6-3sYq30VMc0oGUrZ_XJSWhZ-acRcuZ8L1CEejmWdxBo8PSWwFV9IV2_N-hOszV_Fstg5iMBA and https://zenodo.org/records/16312269?preview=1&token=eyJhbGciOiJIUzUxMiJ9.eyJpZCI6IjEzY2IwMjI1LWI3NjEtNGFmMi1hNzY5LTM5NGI4OWUzYmQ3ZiIsImRhdGEiOnt9LCJyYW5kb20iOiI1M2ZkZDlmNjEyZjlkMjNmZDdmNjhlYWNmYmFlY2YzNSJ9.AzeNBWO2UlekRC_dXMBbKyBULPkFeVWIu8ZL3QwEwHfcW1y9KE02sgpCc2EDV52kJzMaeTiQVX05OcBcE43yNA and is publicly available as of the date of publication.
• Codes generated during this study are available at Github Data: https://github.com/jboulanger/Organoid_morphology and are publicly available as of the date of publication.
• Any additional information required to reanalyze the data reported in this paper is available from the lead contact upon request.
• The published article includes datasets/code generated and analyzed in Chiaradia et al.^1^

## Acknowledgments

We would like to thank members of the Lancaster Laboratory and the Light Microscopy Facility of the MRC Laboratory of Molecular Biology. This work was supported by the 10.13039/501100000265Medical Research Council (MC_UP_1201/9) and the 10.13039/100010663European Research Council (ERC STG 757710). We thank Yoav Gilad (Gallego Romero et al.^23^) for the generation and donation of the chimpanzee iPSC lines.

## Author contributions

I.C. designed and analyzed the experiments, prepared samples, performed the experiments, and wrote the manuscript. J.B. wrote the image analysis macros. M.A.L. supervised the study and wrote the manuscript. S.B.E. and M.R.L. tested the pipeline on +MG/−MG organoids.

## Declaration of interests

M.A.L. is an inventor on patents related to cerebral organoids, with licensing agreements with STEMCELL Technologies, and is a co-founder of a:head bio.
