# Supplementary material for: Protocol for quality control screening of brain organoid morphology
Source: STAR Protoc. 2026 Mar 13;7(1):104423. doi: 10.1016/j.xpro.2026.104423 (PMC12990332; doi:10.1016/j.xpro.2026.104423)
Supplement: Supplementary file 1 — Document S1. Figures S1–S5. [file mmc1.pdf]

Supplemental Figure S1

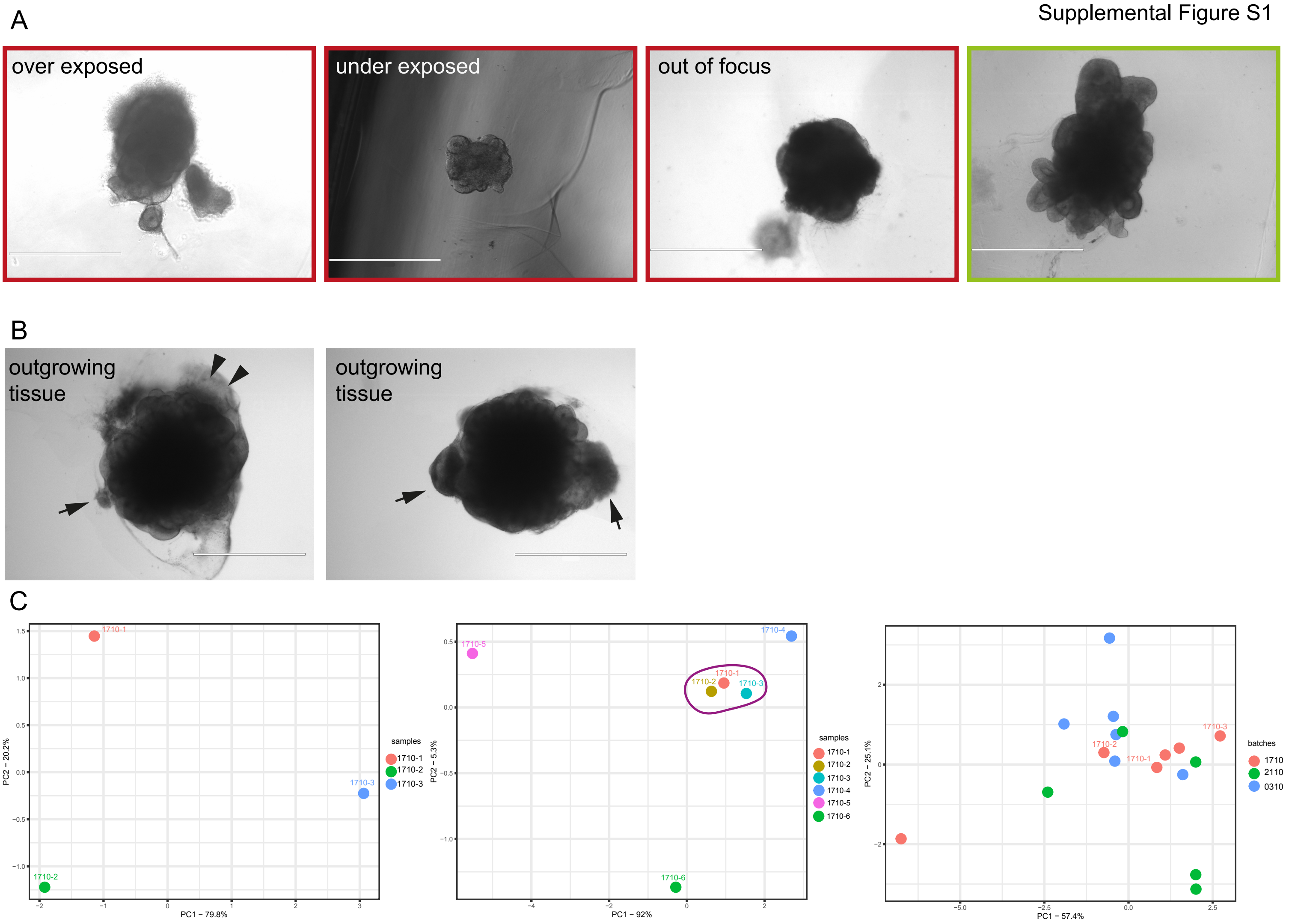

Supplemental Figure S2

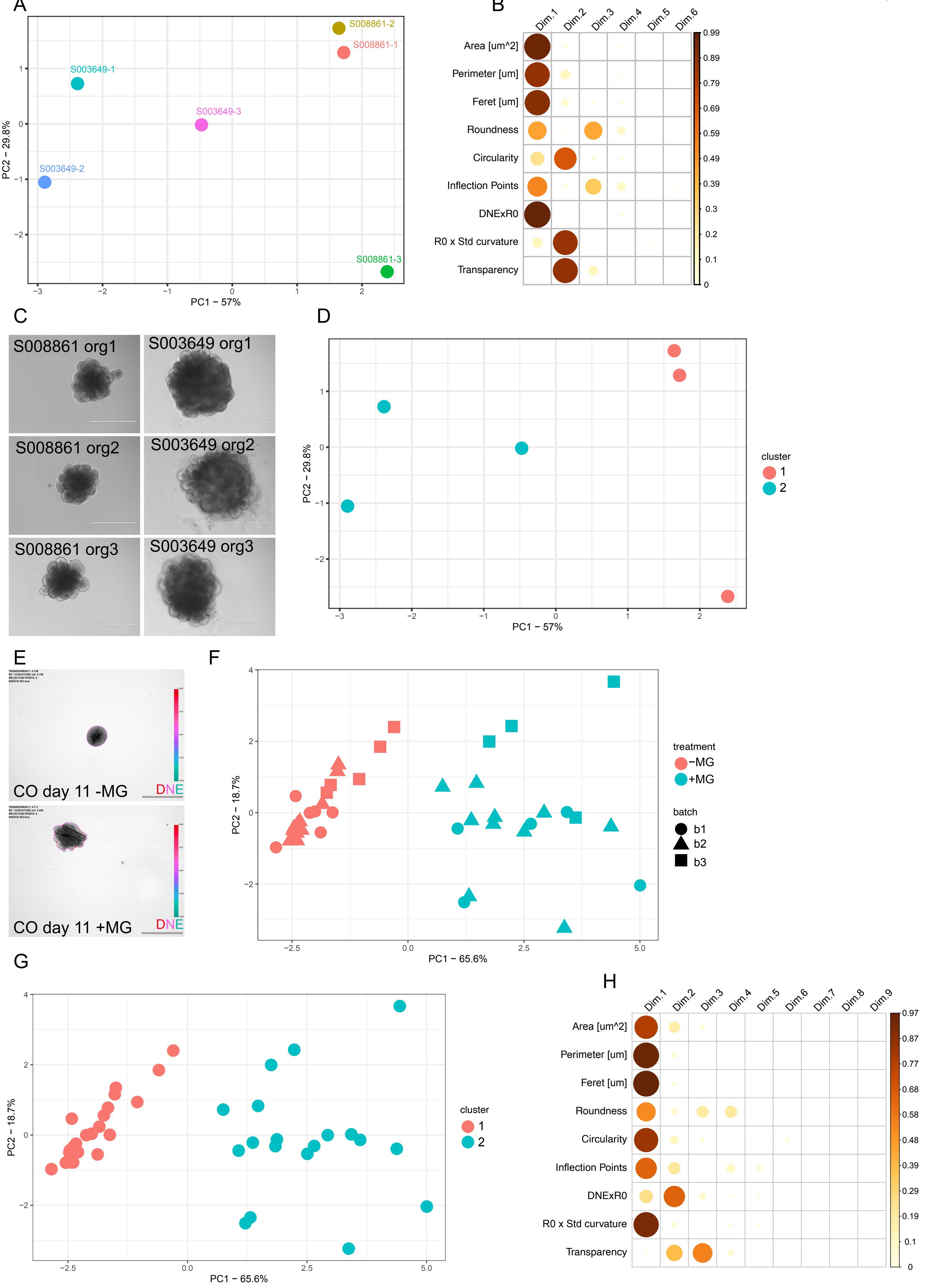

Supplemental Figure S3

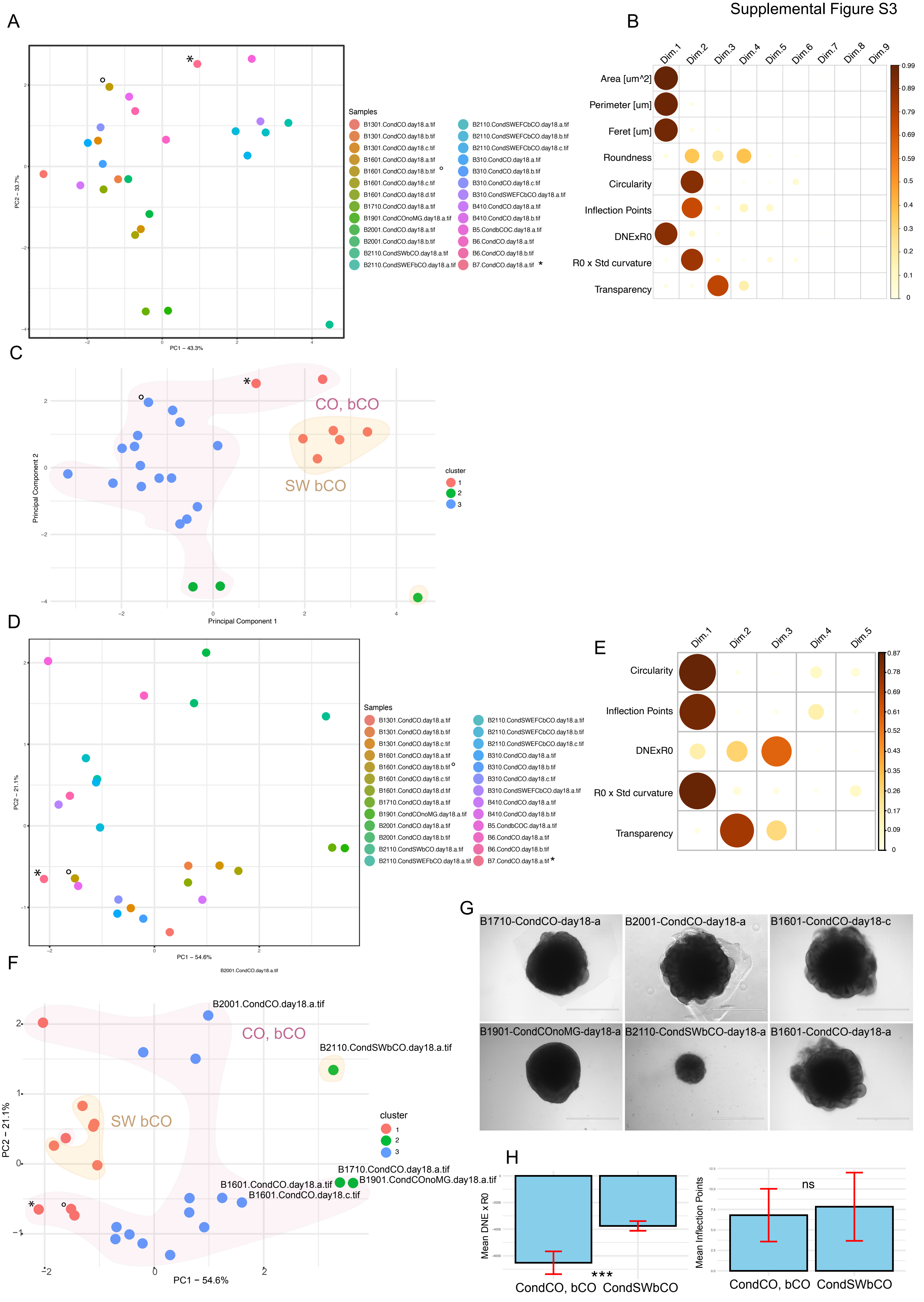

Supplemental Figure S4

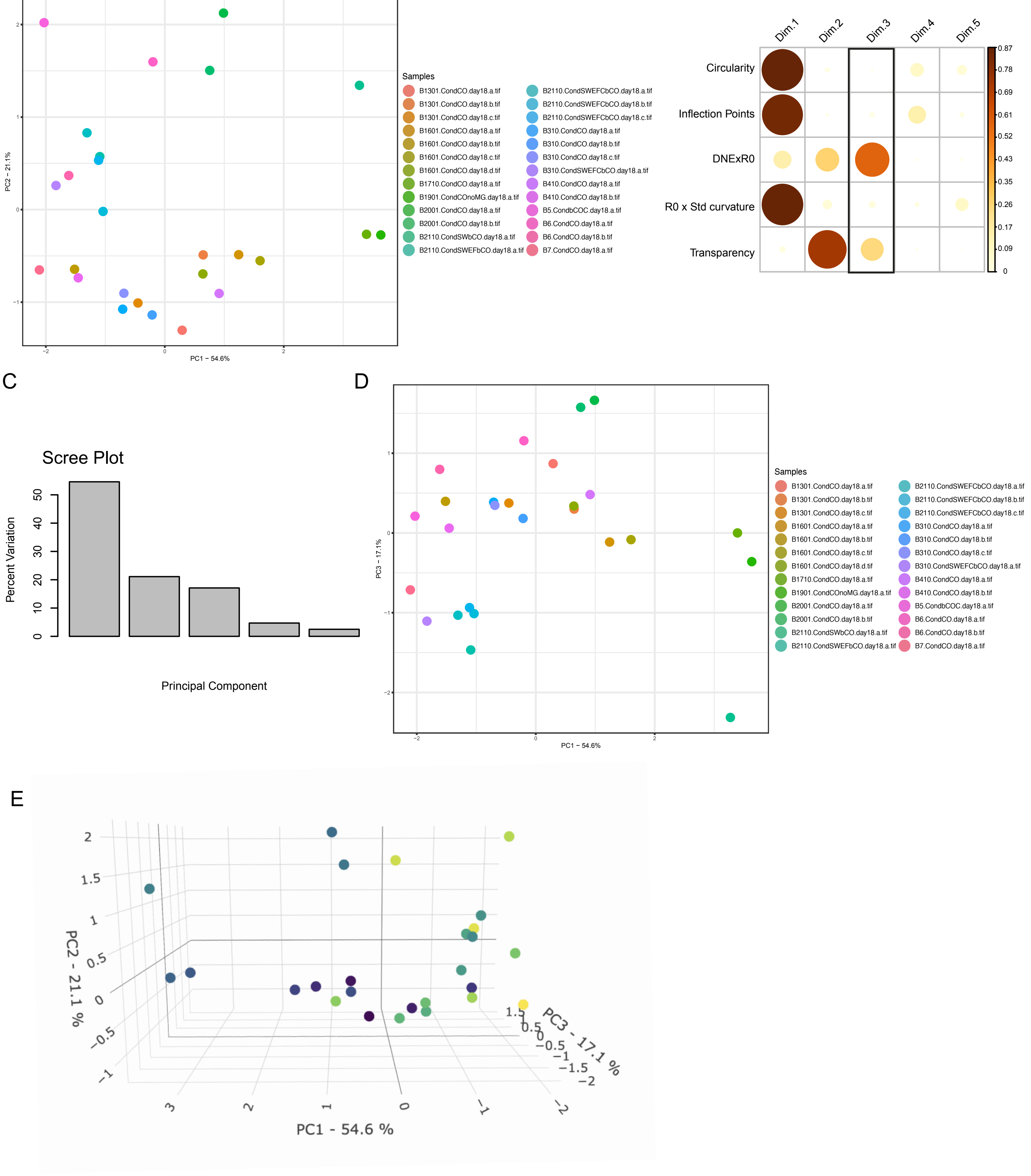

Supplemental Figure S5

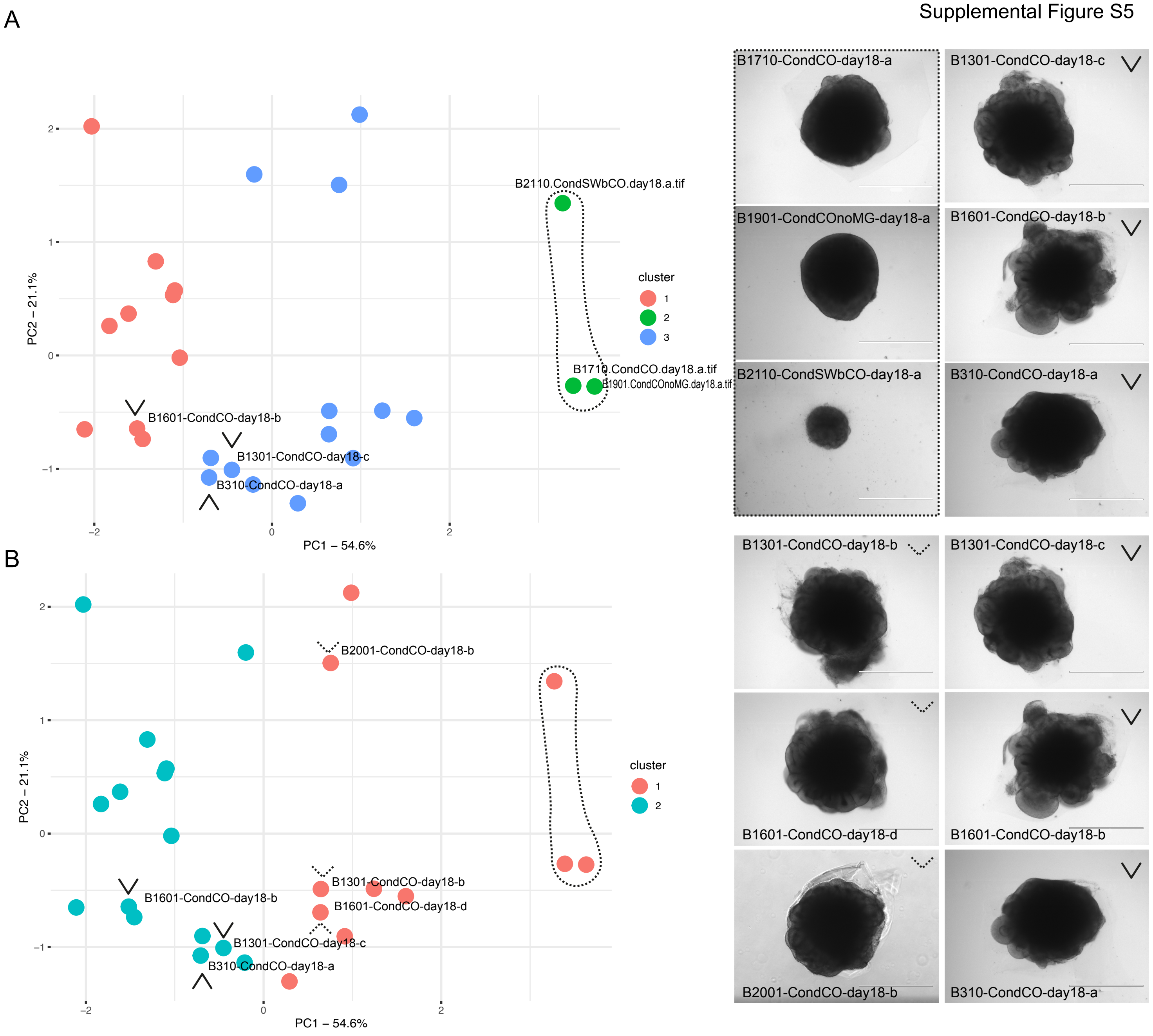

Supplemental Figure S1: Troubleshooting imaging, related to Step 1. A. Examples of out-of-focus, under exposed, over exposed brightfield images from day 18 organoids. Red frames indicate images below quality standard, to be excluded from further analysis. Green frame indicates adequate imaging outcome. Related to Troubleshooting Problem 1. Scale bars: 1000 µm. **B.** Examples of outgrowing tissue from day 18 COs that can interfere with image tracing. Black arrows indicate non-neuroepithelial tissue. Black arrowheads indicate outgrowing neurons. Related to Troubleshooting Problem 2. Scale bars: 1000 µm. **C.** PCA plots showing the distribution along PC1 (x axis) and PC2 (y axis) of n=3 (left plot), n=6 (middle plot) day 18 CO samples from the same batch (B1710). Right PCA shows day 18 COs from three different batches (B1710, B2110, B310). Same-batch organoids can display different distances in the morphospace when samples from other batches are introduced (right plot). Related to Troubleshooting Problem 3.

Supplemental Figure S2: Morphological analysis on chimpanzee iPSC-derived organoids and organoids with Matrigel treatment, related to Steps 5-9. A. Morphospace of six organoids from two different chimpanzee iPSC lines (S003649, S008861) imaged at day 9. PC1 percent variation on the x-axis and PC2 percent variation on the y-axis. **B.** Correlation plot of the PCA analysis showing the contribution of each morphometric parameter as the squared cosine across principal components. Color intensity and size of the circles are proportional to the contribution of each parameter to the principal components. **C.** Brightfield images of the organoids in A. Scale bars: 400 µm. **D.** PCA plot with K-means cluster annotation of the PCA in A. where k=2. The unbiased clustering is based on batch derivation. **E.** Representative images of day 11 COs from H9 ESCs with and without Matrigel (+MG/-MG). Scale bar indicates DNE (Dirichlet Normal Energy). Scale bars: 1000 µm. **F.** 43 COs from three batches (b1, b2, b3) with and without Matrigel (+MG/-MG) were analysed with our morphological pipeline and plotted in the PCA morphospace. Individual samples are color coded based on batch. PC1 percent variation on the x-axis and PC2 percent variation on the y-axis. **G.** PCA plot with unbiased K-means cluster annotation of the PCA in F. where k=2. Cluster annotation matches +MG/-MG treatment. **H.** Correlation plot of the PCA analysis in F. showing the contribution of each morphometric parameter as the squared cosine across principal components. Color intensity and size of the circles are proportional to the contribution of each parameter to the principal components. CO – cerebral organoids made according to an optimized protocol with a proprietary media but lacking patterning molecules.

Supplemental Figure S3: Choosing image analysis parameters, related to Step 7. A, B. Morphospace of the reference dataset with input samples analysed in this paper and the respective correlation plot (B). Correlation plot showing the contribution of each morphometric parameter as the squared cosine across principal components. Color intensity and size of the circles are proportional to the contribution of each parameter to the principal components. **C.** Cluster analysis with k=3 clusters as in Figure 2F. Cluster 2, with organoids below the morphological quality standard (B2110 CondSWbCO day18 a, B1710 CondCO day18 a, B1901 CondCOnoMG day18 a), are separated from the reference dataset along PC2. Purple contour highlights unguided CO and bCO organoids. Yellow contour highlights guided SWbCO with and without EGF/FGF/CHIR. SWbCOs are separated from CO, bCO cluster along PC1. Related to Troubleshooting Problem 3. **D, E, F.** PCA plot with the respective correlation plot (**e**) and cluster analysis (**f**) of the same dataset as in (a-c) but the morphological parameters related to organoid size (Area, Perimeter, Feret's diameter, Roundness) have been excluded from the analysis. Cluster 2, with organoids below the morphological quality standard (B2110 CondSWbCO day18 a, B1710 CondCO day18 a, B1901 CondCOnoMG day18 a), are separated from the reference dataset along PC1. Purple contour highlights unguided CO and bCO organoids. Yellow contour highlights guided SWbCO with and without EGF/FGF/CHIR. Good morphology SWbCOs are embedded in CO, bCO cluster with no separation along PC1. **G.** Brightfield images of organoids below the morphological quality standard and borderline organoids (labels in F). Scale bars: 1000 µm. **H.** Barplots showing individual morphological parameters (DNE x R0, Inflection points) measured as Mean, SD displayed as error bars. Dataset is the same as in the PCA plots in (A-F). Two batches, 6 organoids were analysed for CondSWbCO (including SWEFbCO, SWEFCbCO, SWbCO). Ten batches, 20 organoids were analysed for CondCO (including CO, COnoMG), bCO.

T Mann-Whitney test was performed. P-value ***=<0.001. Related to Troubleshouting Problem 3. Scale bars: 1000 µm. CO – cerebral organoids made according to an optimized protocol with a proprietary media but lacking patterning molecules; CO noMG – CO without the addition of Matrigel; SWbCO – guided cerebral organoid treated with SMAD WNT (TGFß and WNT) inhibitors; bCO – unguided cerebral organoids in basal medium; EFC – EGF, FGF, CHIR.

Supplemental Figure S4: Troubleshooting principal component analysis, related to Step 9. A. PCA analysis of the reference dataset plus input organoids as in Supplemental Figure S3 where organoid size descriptors have been excluded. **B.** Correlation plot with black frame highlighting the contribution of DNE x R0 predominantly in dimension 3 (PC3). **C.** Screeplot showing the contribution of each principal component to the variance of the dataset. **D.** PCA plot as above with PC1 on the x axis and PC3 on the y axis. **E.** 3D PCA plot showing PC1, PC2, PC3 with their respective percentage variation. Related to Troubleshooting Problem 4.

Supplemental Figure S5: Troubleshooting cluster analysis, related to Step 14. A. K-means clustering with k=3 of the dataset in Supplemental Figure S3. Cluster 2 (in green) contains only input organoids with poor morphology (dotted line and respective brightfield images with dotted line contour on the right). Reference organoids are split among cluster 1 (pink) and 3 (aquamarine). Datapoints and their respective brightfield images are shown on the plot with arrowheads. **B.** PCA plot with K-means analysis with k=2. Cluster 1 (pink) contains both input organoids with poor morphology (dotted contour) and reference organoids with borderline morphology (dotted arrowheads). Other reference organoids with good morphology fall in cluster 2 (aquamarine) and are shown by arrowheads. Related to Troubleshooting Problem 5. Scale bars: 1000 µm. CO – cerebral organoids made according to an optimized protocol with a proprietary media but lacking patterning molecules; CO noMG – CO without the addition of Matrigel; SWbCO – guided cerebral organoid treated with SMAD WNT (TGFß and WNT) inhibitors; bCO – unguided cerebral organoids in basal medium; EFC – EGF, FGF, CHIR.
